# Molecular Mechanisms Involved in Schwann Cell Plasticity

**DOI:** 10.3389/fnmol.2017.00038

**Published:** 2017-02-17

**Authors:** Angélique Boerboom, Valérie Dion, Alain Chariot, Rachelle Franzen

**Affiliations:** ^1^GIGA-Neurosciences, University of LiègeLiège, Belgium; ^2^GIGA-Molecular Biology of Diseases, University of LiègeLiège, Belgium; ^3^Walloon Excellence in Lifesciences and Biotechnology (WELBIO)Wavre, Belgium

**Keywords:** Schwann cell, plasticity, molecular mechanisms, nerve injury, peripheral neuropathy

## Abstract

Schwann cell incredible plasticity is a hallmark of the utmost importance following nerve damage or in demyelinating neuropathies. After injury, Schwann cells undergo dedifferentiation before redifferentiating to promote nerve regeneration and complete functional recovery. This review updates and discusses the molecular mechanisms involved in the negative regulation of myelination as well as in the reprogramming of Schwann cells taking place early following nerve lesion to support repair. Significant advance has been made on signaling pathways and molecular components that regulate SC regenerative properties. These include for instance transcriptional regulators such as c-Jun or Notch, the MAPK and the Nrg1/ErbB2/3 pathways. This comprehensive overview ends with some therapeutical applications targeting factors that control Schwann cell plasticity and highlights the need to carefully modulate and balance this capacity to drive nerve repair.

## Introduction

The peripheral nervous system (PNS) shows a surprising capacity of regeneration compared to the central nervous system (CNS). This ability of peripheral nerves to recover quickly following damage is to a large extent due to the remarkable plasticity of Schwann cells (SCs), the glial cells of the PNS (Jessen et al., 2015). SCs are derived from neural crest cells that differentiate into SC precursors and then into immature SCs between E12 and E15 in mice. Around birth, axonal sorting and myelination start in the peripheral nerves. Some SCs establish a 1:1 relationship with large-diameter axons, wrap them multiple times to form a thick and compact myelin sheath. Myelin-forming SCs allow the fast conduction of action potentials by insulating the axons. The non-myelinating SCs typically associate with several small-diameter axons to form Remak bundles. Nerve homeostasis, trophic support and myelin maintenance are other important functions of SCs in adulthood (Jessen and Mirsky, 2005).

Besides their major roles in normal nerve physiology, SCs play a key function for repair in many pathological conditions thanks to their striking plasticity (Zochodne, 2008). For example, after a peripheral nerve injury, they are capable of switching into a SC immature-like phenotype that drives nerve repair. Over the last decades, major progress has been made in unraveling molecular mechanisms and signaling pathways that drive SC dedifferentiation and regulate their plasticity. In this review, we will update and discuss the recent studies that identified molecular components involved in SC plasticity and their possible therapeutic implications.

## Remarkable plasticity and regenerative properties of Schwann cells after peripheral nerve injury

**Figure 1:**
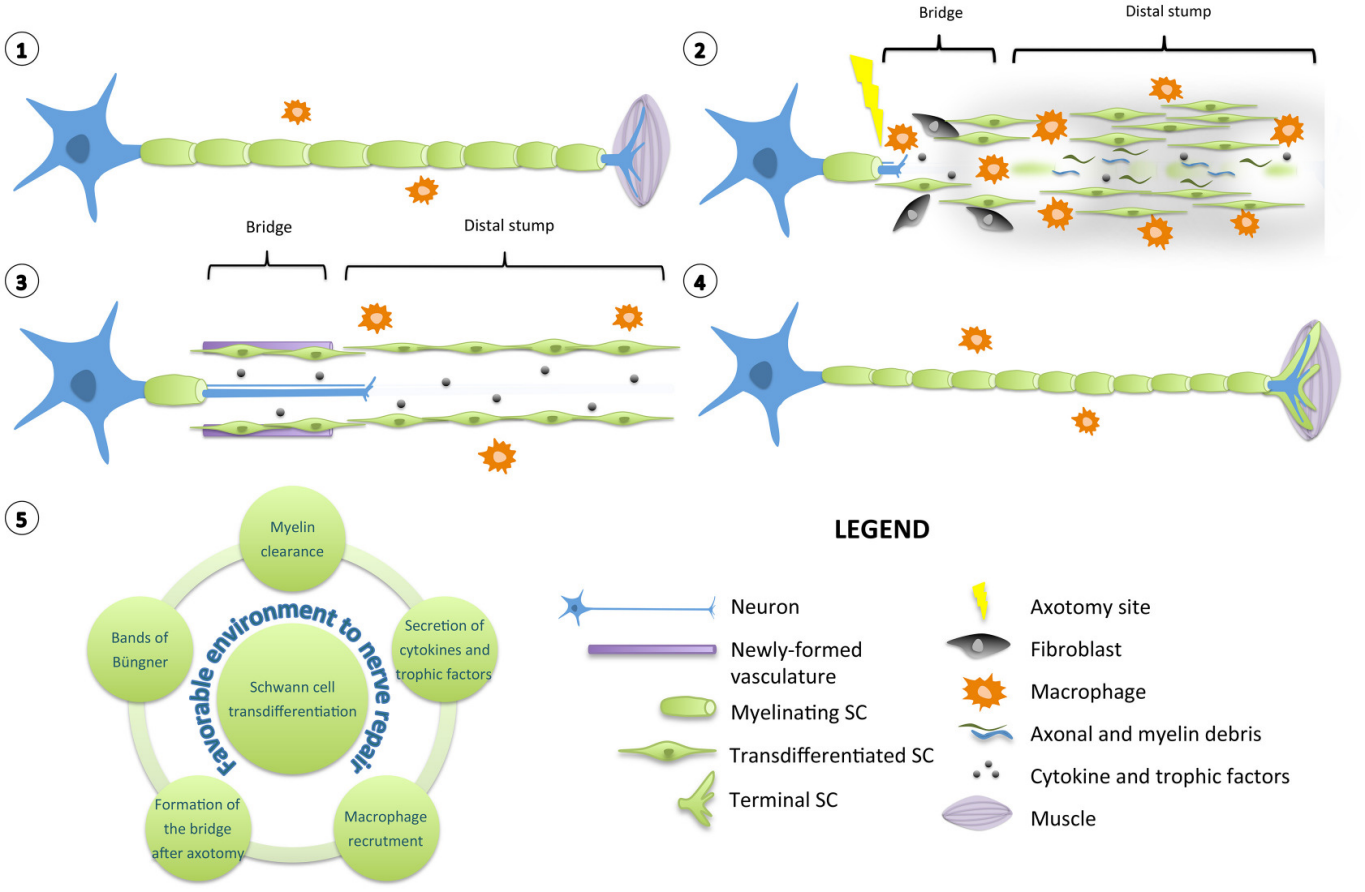
**Diagram of Schwann cell response to nerve injury**. (1) Schematic representation of a single neuron with myelinating SCs and resident macrophages. For simplification, the basal lamina around SCs is not shown. (2) After injury, the nerve distal to the injury site degenerates and undergoes a series of complex multicellular and molecular events in which SCs play a role of orchestrator. SCs transdifferentiate into repair-promoting cells, creating a permissive and favorable environment for nerve regeneration. SCs downregulate pro-myelinating genes and clear their myelin sheaths. They proliferate, secrete several pro-inflammatory cytokines and trophic factors that support glial and neuronal survival and regrowth. Axonal and myelin debris are also phagocyted by resident and blood-derived macrophages recruited by SCs. SCs interact with fibroblasts to build a bridge between the two stumps of the nerve over the lesion site. (3) Newly formed vasculature guides the SCs and the growing axons through the lesion site. In the distal stump, SCs align in tracts named bands of Büngner to provide a trophic and physical support for axons to regrow. (4) After axonal regeneration, transdifferentiated SCs readily exit the cell cycle, differentiate again and remyelinate the axons to support the complete functional recovery. However, the newly-formed myelin sheaths are most of the time shorter and thinner than expected based on axonal diameter. Specialized terminal SCs direct reinnervation by helping the axons to find their paths toward their initial targets. (5) Diagram displaying the various roles played by a transdifferentiated SC to create a favorable environment for nerve repair.

The unique plasticity of SCs has been extensively studied in transgenic animals after cut or crush injuries. Indeed, following a nerve lesion, quiescent, highly specialized myelinating and non-myelinating SCs reprogram into proliferative progenitor-like repair SCs that drive the entire regeneration process (Chen et al., 2007; Jessen and Mirsky, 2016). The reprogramming of SCs in physiopathological conditions is most of the time defined as dedifferentiation. However, transdifferentiation seems more appropriate, as repair SCs, besides re-expressing immature SC markers, exhibit completely different features (Arthur-Farraj et al., 2012). Indeed, the injury-induced conversion of mature SCs in regeneration-promoting cells is an active phenomenon. It involves a down-regulation of pro-myelinating genes including early growth response 2 *(Egr-2*, more frequently named *Krox-20*), POU domain class 3 transcription factor 1 *(Pou3f1* or *Oct-6)*, myelin protein zero *(MPZ) or* myelin basic protein *(MBP)* as well as an up-regulation of markers of immature, de-differentiated SCs such as c-Jun, low affinity neurotrophin receptor (p75NTR) or glial fibrillary acidic protein (GFAP) but also specific repair-supportive genes (Jessen and Mirsky, 2008). Following damage, the nerve undergoes a series of complex multicellular and molecular events in which SCs play a role of orchestrator (Figure 1).

Quickly after nerve injury, damaged axons in the distal stump degenerate in an active process called Wallerian degeneration (Waller, 1850). Yet unidentified signals from damaged nerves induce the reprogramming of SCs. These downregulate pro-myelinating genes and start clearing their myelin sheaths through a mechanism of autophagy called myelinophagy (Gomez-Sanchez et al., 2015). Axonal and myelin debris are also phagocyted by resident and blood-derived macrophages recruited by SCs (Hirata and Kawabuchi, 2002; Lee et al., 2006; Barrette et al., 2008). An inflammatory reaction occurs: many blood cells invade the lesion site and secrete numerous cytokines and chemokines (Martini et al., 2008; Gaudet et al., 2011; Rotshenker, 2011). Following nerve axotomy, particularly, the basal lamina of SCs and the connective tissue are interrupted (Zochodne, 2008). A tissue bridge is formed between the two stumps of the nerve over the lesion site. Fibroblasts play a major role in building this bridge by interacting with SCs (Parrinello et al., 2010). Newly formed vasculature is also crucial to guide the SCs and the growing axons through the lesion site (Cattin et al., 2015). Many chemical and physical interactions happen between the actors present in the injured nerve, creating a permissive and favorable environment for regeneration (Cattin and Lloyd, 2016).

Irrespective of whether the injury is a crush or a cut, repair SCs in the distal stump proliferate, secrete several trophic factors that support glial and neuronal survival and regrowth including artemin, brain-derived neurotrophic factor (BDNF) or glial cell line-derived neurotrophic factor (GDNF) (Fernandez-Valle et al., 1995; Kim et al., 2000; Boyd and Gordon, 2003; Fontana et al., 2012). They also align in tracts named bands of Büngner and provide a trophic and physical support for axons to regrow and reinnervate correctly their targets (Weinberg and Spencer, 1978; Stoll and Müller, 1999). At the neuromuscular junction, specialized terminal SCs direct reinnervation by helping the axons to find their paths toward their appropriate sites (Son and Thompson, 1995). After axonal regeneration, repair SCs readily exit the cell cycle and differentiate again into myelinating and non-myelinating SCs to support the complete functional recovery. Nevertheless, most of the time, the newly-formed myelin sheaths are shorter and thinner than expected based on axonal diameter (Schröder, 1972).

## Molecular mechanisms that modulate Schwann cell plasticity

**Figure 2:**
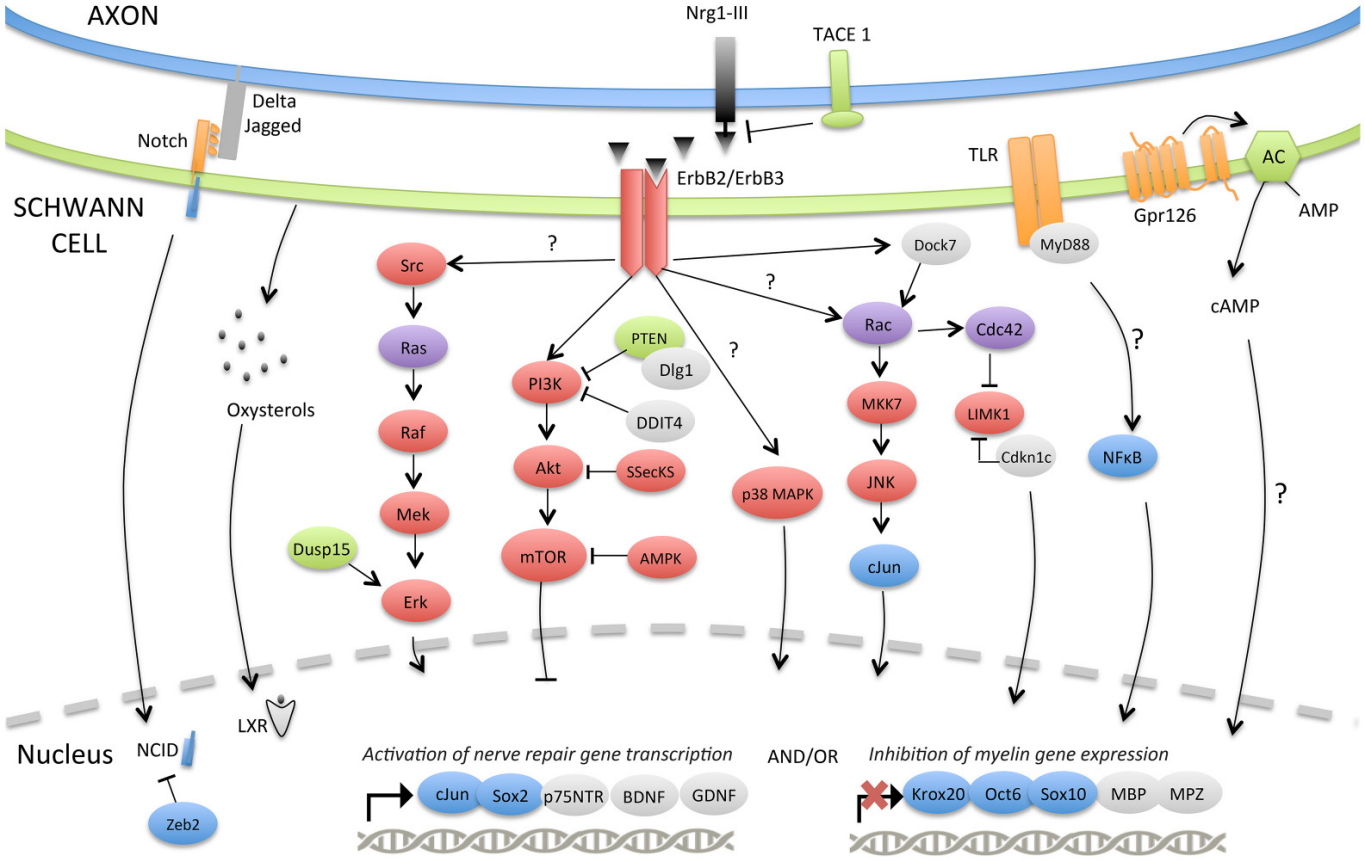
**Molecular signaling pathways involved in SC plasticity, negative regulation of myelination and nerve repair**. The injury-induced reprogramming of SCs in regeneration-promoting cells involves a down-regulation of pro-myelinating genes including *Krox-20, Oct-6, Sox10, MPZ, or MBP* as well as an up-regulation of markers characteristic of immature, de-differentiated SCs such as c-Jun and p75NTR but also specific repair-supportive proteins like BDNF or GDNF. The signaling pathways or molecular components activating (c-Jun, Notch, MAPKs, TLRs, Gpr126, NF-κB) or inhibiting (PI3K/Akt/mTOR) this reprogramming are represented. Some negative regulators of myelination during development (TACE1, LXRs, Dusp15, PTEN, Dlg1, DDIT4, Dock7, Zeb2) and possibly playing a role after injury are also shown.

The adaptive reprogramming of SCs after a nerve injury is a prerequisite for regeneration. Therefore, understanding the molecular components that regulate the phenotypic change of adult specialized SCs into repair-supportive SCs can lead to the development of new therapeutics that can boost repair in various peripheral neuropathies. In this section, we will review the most relevant molecular components or pathways that have been identified for controlling SC plasticity, the negative regulation of myelination and their response to nerve injury (Tables 1, 2; Figure 2).

**Table 1:** **Most relevant molecular components involved in Schwann cell plasticity, negative regulation of myelination and nerve repair**.

**Molecular component**	***In vivo* model(s)**	**Phenotype(s)**	**Reference(s)**
**TRANSCRIPTIONAL REGULATORS**			
c-Jun	MPZ^Cre^/cJun^fl/fl^	Injury: lack of trophic factor expression, no alignment in bands of Büngner, no axon regeneration and no functional recovery	Parkinson et al., 2008; Arthur-Farraj et al., 2012; Fontana et al., 2012
Notch	MPZ^Cre^/RBPJ^fl/fl^;Notch^fl/fl^ ; NCID^CASL−STOPfl/fl^ Rats treated with Jagged1 (Notch signaling activator)	Development: acceleration of myelination and thicker myelin. Injury: delayed myelin breakdown Injury: improved axonal regeneration and functional recovery	Woodhoo et al., 2009; Wang et al., 2015
Zeb2	PLP^CreERT2^/Zeb2^fl/fl^	Injury: disturbed regeneration and lack of remyelination	Quintes et al., 2016; Wu L. M. N. et al., 2016
NF-κB	GFAP-IκBα-dn	Injury: delayed axonal regeneration and disturbed remyelination	Morton et al., 2012
**MAPK**			
Raf/Erk	MPZ^RafTR^ (tamoxifen-inducible Raf activation)	Normal adult nerve: transient activation leads to demyelination, SC proliferation and increased P75NTR expression. Sustained activation leads to inflammation.	Napoli et al., 2012
Rac/JNK	Mice transfected with dominant negative Rac1/Rac1-RNAi Mice injected with Rac inhibitor Dock7 shRNA transgenic mice (decrease of Rac/JNK signaling)	Injury: reduced myelin sheath fragmentation Injury: decreased c-Jun and p75NTR expression Development: enhanced myelin thickness	Jung et al., 2011; Yamauchi et al., 2011; Shin et al., 2013
p38	Mice injected with p38 inhibitor MPZ^Cre^/p38a^fl/fl^ New-born rats treated with BMP7 (activation of p38MAPK signaling)	Injury: reduced SC demyelination and dedifferentiation Development: acceleration of myelination Injury: delay in myelin clearance, small increase of re-myelination Development: delayed peripheral myelination	Yang et al., 2012; Liu et al., 2016b; Roberts et al., 2016
**PI3K/Akt/mTOR**			
PTEN	PLP^CreERT2^/PTEN^fl/fl^ (hyper-activation of PI3K/Akt signaling) PLP^CreERT2^/PTEN^fl/fl^treated with rapamycin (mTOR inhibitor)	Development: hypermyelination, myelin outfoldings Amelioration of the myelin pathology	Goebbels et al., 2010, 2012
Dlg1	Mice injected with Dlg1 shRNA (activation of PI3K/Akt signaling) MPZ^Cre^Dlg1^fl/f^ (activation of PI3K/Akt signaling)	Development: myelin outfoldings and peripheral nerve overmyelination Development: transient hypermyelination	Cotter et al., 2010; Noseda et al., 2013
DDIT4	DDIT4 KO mouse (over-activation of PI3K/AKT/mTOR signaling)	Development: sustained hypermyelination	Noseda et al., 2013
**WNT SIGNALING AND LXR**			
LXR	LXR KO mouse Mice treated with paraquat (LXR activation)	Development: altered myelination Development: severe myelin sheath disorganization	Makoukji et al., 2011; Hichor et al., 2016
**TLR SIGNALING**			
TLRs	TLR2, TLR4, MyD88 KO mice	Injury: impaired expression of inflammatory modulators, macrophage recruitment and activation, axonal regeneration and functional recovery	Boivin et al., 2007
**GPCR SIGNALING**			
Gpr126	PLP^CreERT2^/Gpr126^fl/fl^	Injury: defects in remyelination, macrophage recruitment and axon regeneration	Mogha et al., 2016b
**Nrg1/ErbB2-B3**			
Nrg1	Animals treated with Nrg1 isoforms Inactivation in axons: SLICK-A^Cre^; CAG^CreERT2^/Nrg1^fl/fl^ Inactivation in SCs: Dhh^Cre^/Nrg1^fl/fl^	Injury: improved nerve regeneration and functional recovery Injury: slower axon regeneration, target reinnervation, remyelination, functional recovery Injury: impaired remyelination	Chen et al., 1998; Joung et al., 2010; Fricker et al., 2011, 2013; Yildiz et al., 2011; Stassart et al., 2013
BACE1	BACE1 KO mouse	Injury: impaired remyelination but enhanced axon regeneration	Hu et al., 2008; Farah et al., 2011
TACE	HB9^Cre^/TACE^fl/fl^	Development: hypermyelination	La Marca et al., 2011
ErbB2	Mice treated with ErbB2 inhibitor Plp1^CreERT2^ErbB2^fl/fl^	Injury: decreased demyelination Injury: no effect on SC proliferation and survival	Guertin et al., 2005; Atanasoski et al., 2006

**Table 2:** **Summary of the regulators and their roles in SCs**.

**Negative regulation of myelination during development**	**Positive regulation of myelination during development**
c-Jun, Notch, Sox2, Pax-3, Id2, Egr-1, Egr-3, Cdkn1c, Hes5, Dock7, Cthrc1, p38MAPK, BMP7, SSeCKS, Cadm3, Dlg1, PTEN, DDIT4, AMPK, LXR?	Zeb2, NF-κB?, PI3K/Akt/mTOR, LXR?, Wnt, Gpr126, Gpr44, LPA1, Nrg1/ErbB2/3
**Transdifferentiation after injury**	**Remyelination after injury**
c-Jun, Notch, NF-κB?, Sox2, Ras/Raf/Erk, Rac/JNK, p38 MAPK, Sam68, Dixdc1, TLRs, Gpr126, Nrg1/ErbB2/3?	Zeb2, NF-κB?, Nrg1/ErbB2/3

### Transcriptional regulators

#### The transcription factor c-Jun

The transcription factor c-Jun does not appear to be essential during SC development but multiple arguments demonstrate its central function in SC reprogramming. Firstly, the time course of c-Jun expression supports its role in SC plasticity. Indeed, c-Jun is down-regulated post-natally during SC differentiation and myelination and is highly up-regulated under pathological conditions such as peripheral nerve injuries, demyelinating diseases or other peripheral neuropathies (Stewart, 1995; Parkinson et al., 2008; Hutton et al., 2011; Hantke et al., 2014; Klein et al., 2014). Secondly, c-Jun is a negative regulator of myelination and a cross-antagonist of Krox-20, one of the main pro-myelinating transcription factors. Deletion of c-Jun in SCs *in vitro* strikingly facilitates Krox-20-induced expression of MBP or MPZ. Conversely, forced expression of c-Jun inhibits the expression of myelin proteins. Moreover, the expression of c-Jun and Krox-20 is mutually exclusive: while immature SCs express relatively high levels of c-Jun and low levels of Krox-20, myelinating cells switch to high levels of Krox-20 and low levels of c-Jun (Parkinson et al., 2004, 2008). Thirdly, strong evidence *in vivo* exhibits the involvement of c-Jun in major aspects of the SC response following nerve injury. Myelination during development occurs normally in the c-Jun conditional knockout mice (Parkinson et al., 2008). However, the absence of c-Jun following a nerve lesion results in a failure of generating the repair SCs, leading to impaired axonal regeneration, target reinnervation and functional recovery. The myelin sheath degradation and clearance are also strikingly slowed in the absence of c-Jun *in vitro* and *in vivo*. Moreover, mutant SCs fail to form the Büngner regenerative tracks and to express the adhesion molecules and trophic factors critical for nerve repair (Parkinson et al., 2008; Arthur-Farraj et al., 2012; Fontana et al., 2012).

The role of c-Jun in SCs after nerve injury is to activate a repair program to support regeneration. The high levels of c-Jun found in different peripheral neuropathies that do not involve nerve injuries like Charcot-Marie Tooth (CMT) raises the question on the precise function of c-Jun in these diseases. Does c-Jun counteract the pathology, attenuate its symptoms and promote repair or does its up-regulation cause the disease? In two models of demyelinating diseases, CMT1A and CMT1X, c-Jun is increased in SCs that retain myelin differentiation (Hantke et al., 2014; Klein et al., 2014). The myelin sheaths are also relatively normal in a mouse mutant showing a 5–8 fold c-Jun overexpression in SCs (levels that are lower than those seen after a nerve cut though) (Jessen and Mirsky, 2016). Moreover, c-Jun and repair-associated genes are up-regulated in a mouse model in which the serine/threonine-protein kinase (LKB1, also called STK11) is inactivated in SCs and in which axons are damaged without overt demyelination (Beirowski et al., 2014). This suggests that moderated c-Jun up-regulation is compatible with myelination, does not necessarily provoke demyelination and that it is sufficient to activate the transcription of injury-related genes (Jessen and Mirsky, 2016). In this context, it is likely that the role of c-Jun in those diseases is to promote neuroprotection and repair. However, if c-Jun is activated in an uncontrolled and exacerbated manner, c-Jun could also be detrimental. For example, the increase of c-Jun expression in some human schwannomas may directly or indirectly be associated with the abnormal proliferation and the absence of myelin markers (Shivane et al., 2013).

#### Notch signaling

Neurogenic locus notch homolog protein (Notch) is a transmembrane receptor protein that, upon ligand binding, is cleaved and generates an intracellular domain (NICD), which acts as a transcriptional regulator. Notch controls SC proliferation and promotes the generation of immature SCs from Schwann cell precursors (SCPs) *in vivo* but also acts as a negative regulator of myelination. It is down-regulated as myelination proceeds and its inactivation or over-activation leads to premature or delayed myelin formation, respectively. Moreover, the inhibition of Notch signaling in adult mice decelerates myelin breakdown following a nerve lesion (Woodhoo et al., 2009). The addition of recombinant jagged1, an activator of Notch, in rat nerves after injury improves nerve regeneration and functional recovery (Wang et al., 2015). This indicates that stimulation of Notch signaling in SCs could represent an interesting therapeutic strategy to efficiently promote nerve repair. However, aberrant activation of Notch signaling in SCs may also participate to the development of malignant peripheral nerve sheath tumors (Li et al., 2004).

#### The transcriptional repressor Zeb2

More recently, the function of a transcriptional repressor, Zeb2, has been investigated in SC myelination and nerve repair by two different teams (Quintes et al., 2016; Wu L. M. N. et al., 2016). They both showed that mice lacking Zeb2 in SCs develop a severe peripheral neuropathy, caused by a failure of SC maturation. Zeb2 transcriptional repression of negative regulators of myelination such as Notch and SRY-related HMG-box gene 2 (Sox2) is necessary for SC lineage progression. Although Zeb2 seems dispensable for the establishment of the repair SCs following nerve injury, its absence disrupts nerve regeneration and remyelination (Quintes et al., 2016). Mechanistically, Zeb2 controls SC differentiation and remyelination by recruiting histone deacetylases 1 and 2 (HDAC1/2) and NuRD complexes and by inhibiting a Notch–Hey2 signaling axis (Wu L. M. N. et al., 2016).

#### NF-κB

The nuclear factor κB (NF-κB) transcription factor regulates a lot of physiological processes and mediates the physiopathological inflammatory response in many diseases. Several studies suggest that NF-κB activation is essential for the differentiation and myelination of SCs *in vitro* (Nickols et al., 2003; Yoon et al., 2008; Limpert and Carter, 2010). Furthermore, it has been shown that the deacetylation of NF-κB mediated by HDAC 1/2 is necessary to control myelination in the PNS (Chen et al., 2011). Paradoxically, by using transgenic mice in which NF-κB activation is inhibited in SCs, Morton and colleagues demonstrated that NF-κB is dispensable for developmental myelination *in vivo* (Morton et al., 2013). However, they showed that the inhibition of NF-κB signaling in SCs following sciatic nerve crush transiently delays axonal regeneration and remyelination (Morton et al., 2012). This highlights again the differences between immature SCs during development and transdifferentiated repair-SCs. Importantly, NF-κB activity is robustly enhanced after peripheral nerve damage and in inflammatory neuropathies (Andorfer et al., 2001; Laura et al., 2006; Smith et al., 2009; Fu et al., 2010). The different NF-κB-induced transcriptional targets responsible for SC beneficial effects after nerve injury still have to be determined. Of note, the placental growth factor (PlGF) which is up-regulated by NF-κB in SCs following injury, is critical for SC proliferation, myelin sheath degradation, macrophage invasion and axonal regeneration (Chaballe et al., 2011). Targeting NF-κB or its downstream targets in SCs may offer new therapeutic strategies for PNS regeneration. Nevertheless, it is essential to keep in mind that aberrant activation of NF-κB may also lead to schwannoma (Ammoun et al., 2014; Dilwali et al., 2015).

#### Sox-2, Pax-3, Id2, Egr-1, Egr-3

Other factors that regulate gene transcription have been studied in SC dedifferentiation. These are most of the time negative regulators of myelination that functionally counteract positive regulators of myelination including Krox-20 or Oct-6 and are likely to have major roles after nerve damage (Jessen and Mirsky, 2008). For instance, the transcription regulators Sox-2, paired box protein 3 (Pax-3), DNA-binding protein inhibitor 2 (Id2) and early growth response proteins 1 and 3 (Egr-1 and Egr-3) could represent good candidates for regulating SC dedifferentiation in damaged nerves. As for c-Jun or Notch, they are inactive or expressed at low levels in myelinating cells and re-expressed at high levels under conditions that lead to SC dedifferentiation such as nerve injury (Kioussi et al., 1995; Nikam et al., 1995; Stewart et al., 1997; Le et al., 2005; Parkinson et al., 2008; Doddrell et al., 2012). *In vitro*, Sox2, Pax3, and Id2 decrease or increase myelin gene expression when overexpressed or depleted, respectively (Kioussi et al., 1995; Mager et al., 2008; Parkinson et al., 2008; Doddrell et al., 2012). Egr1 and Egr3 are both necessary to up-regulate p75NTR in SC primary cultures, known to be re-expressed in SCs following a nerve injury (Nikam et al., 1995; Topilko et al., 1997; Gao et al., 2007). It remains to be unraveled by *in vivo* evidence whether those different factors are required for SC response after nerve injury.

Besides its likely role in SC plasticity, it has been shown that Sox-2 is necessary in SCs for the formation of a nerve bridge after a complete transection. This process is mediated by an ephrin-B/EphB2 signaling between fibroblasts and SCs that initiates cell sorting, followed by directional collective cell migration of SCs out of the nerve stumps to guide axons across the injury wound (Parrinello et al., 2010).

### MAPK signaling

A growing number of studies provide evidence on the functions of mitogen activated protein kinase (MAPK) family proteins in the regulation of SC plasticity. Extracellular signal regulated kinase (Erk), c-jun N-terminal kinase (JNK) and p38 MAP kinase are rapidly and highly activated in the SCs of the distal stump following a nerve lesion and play specific, overlapping or complementary roles in the SC response after injury (Sheu et al., 2000; Harrisingh et al., 2004; Zrouri et al., 2004; Agthong et al., 2006; Parkinson et al., 2008; Lee et al., 2014; Ronchi et al., 2016).

#### Ras/Raf/Erk

Harrisingh and coworkers were the first ones to demonstrate the role of the Ras/Raf/Erk signaling in SC dedifferentiation (Harrisingh et al., 2004). They showed that the ectopic activation of Raf, an upstream activator of Erk, suppresses the differentiation of primary SCs induced by cyclic adenosine monophosphate (cAMP). Raf activation in cultured differentiated SCs also drives their dedifferentiation. Importantly, they demonstrated that sustained activation of the Ras/Raf/Erk pathway in myelinated dorsal root ganglion (DRG) neuron-SC co-cultures induces demyelination even in the presence of normal axon signaling. Consistent with this idea, another paper reported that the dual specificity phosphatase 15 (Dusp15) is necessary for full activation of Erk and that its activation represses the expression of several myelin genes including *MBP* (Rodríguez-Molina et al., 2017). While high and sustained activation of Ras/Raf/Erk signaling seems essential for SC plasticity, Ras over-activation might also be deleterious and lead to the development of SC-derived tumors in neurofibromatosis type 1 patients (Harrisingh et al., 2004). A strong activation of Erk has actually been reported in human primary schwannoma cells (Ammoun et al., 2008).

More recently, the strategy of Raf high activation has been used *in vivo* to determine whether this signaling was central in controlling SC plasticity (Napoli et al., 2012). Napoli et al. demonstrated that a tamoxifen-inducible Raf transgene in SCs is sufficient to induce the down-regulation of myelin proteins and the expression of dedifferentiation markers such as p75NTR, even in the absence of axonal damage. The duration of Raf activation, controlled by the period of tamoxifen treatment, determines the time frame of SC demyelination and proliferation. A prolonged tamoxifen treatment and activation of Raf in SCs also induces an inflammatory response, which includes the breakdown of the blood-nerve barrier and the infiltration of macrophages, which promote nerve regeneration.

Although the repression of Erk signaling with specific inhibitors blocks SC mitosis, cytokine expression and demyelination, it only prevents partially the induction of p75NTR or GDNF and the shutdown of myelin gene expression (Napoli et al., 2012; Shin et al., 2013). Thus, even if the sustained and pronounced activation of the Ras/Raf/Erk pathway is fundamental in regulating SC plasticity, this supports the idea that several independent mechanisms control multiple aspects of SC plasticity. Moreover, different studies showed that Erk activation is actually pro-myelinating and that its inhibition blocks SC differentiation and myelination *in vivo* (Grossmann et al., 2009; He et al., 2010; Newbern et al., 2011). Therefore, Erk signaling is necessary both for the differentiation of SCPs and for the dedifferentiation of adult SCs following a nerve injury. The reconciling explanation could be that distinct levels of Erk activity would define the state of SC differentiation. Low or basal activity would be required for SC differentiation while high Erk levels would drive dedifferentiation and proliferation (Napoli et al., 2012; Newbern and Snider, 2012).

Finally, the role of c-Jun as a downstream target of Erk signaling is an attractive idea but it is actually quite controversial and further *in vivo* studies are necessary to better understand the signaling mechanisms downstream of Erk in SC plasticity. Indeed, Syed et al. showed that Erk inhibition abolishes both the demyelination and the expression of c-Jun induced by Neuregulin 1 (Nrg1) treatment in SC-DRG neuron co-cultures (Syed et al., 2010). Additionally, the Lloyd group indicated that c-Jun is strongly up-regulated after Raf over-activation in SCs *in vivo* (Napoli et al., 2012). However, others reported that the induction of c-Jun and SC dedifferentiation triggered by cAMP removal *in vitro* depends on JNK rather than on Erk activity (Monje et al., 2010; Shin et al., 2013). Actually, other data revealed that Rac/JNK and Raf/Erk might be responsible for different complementary functions in SC plasticity. For example, microarray experiments showed that the expression of regeneration-associated genes, including *p75NTR* or *GDNF* is dependent on JNK but not on Erk signaling. In addition, it has been demonstrated that most Erk-dependent genes in dedifferentiated SCs do not require Rac activity (Shin et al., 2013).

#### Rac/JNK

JNK is another MAPK that plays important functions in SCs. It has been demonstrated that c-Jun activation by JNK is essential for SC migration and proliferation *in vitro* (Parkinson et al., 2001, 2004; Yamauchi et al., 2003). Parkinson and colleagues showed that overexpressing the MAPK kinase 7 (MKK7), a protein upstream of JNK, in SC primary cultures, inhibits myelin gene expression by elevating c-Jun, suggesting a role for JNK signaling in SC plasticity (Parkinson et al., 2008). This was confirmed by Monje et al. who revealed that a reduction of cAMP *in vitro* is sufficient to induce SC dedifferentiation through a JNK-dependent mechanism (Monje et al., 2010). More recently, it has also been demonstrated that the active Rac1 GTPase (Rac), an upstream regulator of MKK7 and JNK, inhibits SC differentiation. In SC cultures, Rac up-regulates c-Jun and down-regulates Krox-20 through the MKK7/JNK pathway, but not through the Raf/Erk pathway. Furthermore, MKK7 activation and the induction of c-Jun observed in sciatic nerves after axotomy were blocked by Rac inhibition (Shin et al., 2013). In addition, Rac activation has been observed in the distal stump of injured nerves, and specific Rac inhibition with a dominant negative Rac1 and Rac1-RNAi decelerates myelin sheath fragmentation. Rac would actually promote myelin sheath fragmentation by controlling actin polymerization in the Schmidt-Lantermann incisures of SCs (Jung et al., 2011).

The cyclin-dependent kinase inhibitor 1 (Cdkn1c), also called p57Kip2, is a protein first known to block G_1_/S transition during cell cycle that also regulates actin filament dynamics through the translocation of LIM domain kinase 1 (LIMK1), a downstream effector of Rac/Cdc42/Rho (Yokoo et al., 2003; Heinen et al., 2013). Knocking down Cdkn1c in cultured SCs leads to cell cycle exit, morphological changes and up-regulation of myelin genes. In SC-DRG neuron co-cultures, Cdkn1c depletion accelerates myelination. A global gene expression analysis also revealed a shift of the transcriptional expression program toward the pattern of differentiating SCs, strongly suggesting a role for Cdkn1c as an important negative regulator of myelination (Heinen et al., 2008). The same team also showed that Cdkn1c is a direct target of the enhancer of zeste homolog 2 (EZH2) and identify Hes5 as a new transcriptional repressor of myelin genes (Heinen et al., 2012). Indeed, EZH2 binds Cdkn1c promoter and its suppression results in a decrease of histone H3K27 trimethylation, an induction of Cdkn1c expression and a down-regulation of myelin genes in cultured primary SCs.

Yamauchi and co-workers reported that the atypical guanine-nucleotide exchange factor dedicator of cytokinesis protein 7 (Dock7), activates Rac/Cdc42/JNK signaling in SCs to positively regulate migration and negatively regulate differentiation and myelination (Yamauchi et al., 2008, 2011). Indeed, knocking down Dock7 in primary SCs results in an increase of cAMP-mediated differentiation and in a shorter duration of activation of Rac/Cdc42 and JNK while silencing Dock7 in mice increases myelin thickness.

Finally, collagen triple helix repeat containing 1 (Cthrc1) is another protein that has been linked to Rac activation in SCs. Apra and colleagues investigated Cthrc1 function in SCs both *in vitro* and *in vivo* (Apra et al., 2012). They showed that its silencing inhibits SC proliferation and promotes their migration and myelination *in vitro*. By using a transgenic mouse line, they demonstrated that the overexpression of Cthrc1 in SCs leads to a delayed myelination with SCs maintaining a proliferative state. More recently, Zhou et al identified miR-9 as an important negative regulator of SC migration targeting Cthrc1 after nerve injury (Zhou et al., 2014). The up-regulation of miR-9 decreases SC migration in culture and following nerve injury. miR-9 has also been shown to directly target Cthrc1, which in turn inactivates downstream Rac. Finally, miR-9 is down-regulated at a speed comparable to the rate of SC migration during nerve regeneration, suggesting that miR-9 down-regulation could be linked to the phenotypic modulation of SCs after nerve injury.

All these data confirm that Rac/JNK signaling is essential for SC plasticity, proliferation and migration following nerve injury. However, stimulating this pathway for nerve repair therapy must be cautioned as its upregulation has also been observed in primary human schwannoma cells (Kaempchen et al., 2003).

#### p38 MAPK

Yang and co-workers provided the most relevant data that suggest a role for p38 MAPK in SC plasticity and their response to injury (Yang et al., 2012). They reported that inhibition of p38 MAPK activity in mice blocks SC demyelination and dedifferentiation following nerve injury, despite ongoing axon degeneration. In myelinating co-cultures, they also showed that p38 MAPK mediates myelin breakdown induced by Nrg1. Finally, while the ectopic activation of p38 MAPK is sufficient to provoke myelin breakdown and c-Jun induction, its inhibition in SC-DRG neuron co-cultures promotes myelination. Roberts and collaborators recently investigated the *in vivo* function of the major p38 isoform in mouse conditional knockouts. In agreement with previous findings, the lack of p38alpha in SCS accelerates myelination during development, delays myelin clearance and slightly increases remyelination following injury. However, axonal regeneration and functional repair were not affected in the absence of p38alpha (Roberts et al., 2016).

A recent study on bone morphogenetic protein 7 (BMP7) in SCs revealed a role for this protein as a negative regulator of myelination through the activation of p38 MAPK signaling (Liu et al., 2016b). Liu and collaborators showed that BMP7 expression is inversely correlated with myelin gene expression during normal peripheral myelination. In addition, BMP7 treatment decreases cAMP-induced myelin gene expression and up-regulates c-Jun in primary SC cultures. By using p38 MAPK inhibitor, gene silencing and rescue experiments, they reported that p38 MAPK is responsible for BMP7-mediated suppression of myelin gene expression. Finally, when injected in newborn rats, BMP7 retards peripheral myelination.

#### PI3K/Akt/mTOR signaling

Phosphatidylinositol 3-kinase (PI3K) signaling has been implicated in the regulation of proliferation, survival and differentiation of different cell types (Rameh and Cantley, 1999). The pathway is strongly activated in primary cultured SCs following treatment with soluble Nrg1 or contact with neurite membranes. Moreover, PI3K signaling is required for SC proliferation and survival *in vitro* (Maurel and Salzer, 2000; Li et al., 2001). Its inhibition affects the initiation of myelination but is not necessary for myelin maintenance in SC-neuron co-cultures (Maurel and Salzer, 2000). In addition, the selective activation of Akt in SCs by gene transfer results in an increased myelination in SC-DRG neuron co-cultures and allogenic nerve graft experiments. This last paper also demonstrated opposing functions for PI3K/Akt and Erk pathways in controlling SC myelination (Ogata et al., 2004). The imbalanced activation of the PI3K/Akt and Erk pathways has also been revealed in transgenic rodent models of CMT1A in which SCs develop persistent differentiation defects during early postnatal development (Fledrich et al., 2014). The transgenic expression of constitutively active Akt in SCs, however does not seem to disrupt PNS myelination *in vivo* (Flores et al., 2008).

Studies investigating the role of specific proteins indirectly indicated a role for PI3K/Akt signaling in the regulation of SC proliferation and differentiation after nerve injury. For example, Src-associated in mitosis of 68 kD (Sam68) has been suggested to promote SC proliferation by enhancing the PI3K/Akt pathway and to act on regeneration following sciatic nerve crush (Wu et al., 2016b). DIX domain containing-1 (Dixdc1), which is also linked to PI3K/Akt activation, promotes SC proliferation after sciatic nerve crush. Dixdc1 is up-regulated following nerve damage and its depletion leads to reduced PI3K/Akt activation and SC proliferation (Wu et al., 2016a). Consistently, 17β-estradiol has been shown to promote SC proliferation and early remyelination in the bridge after nerve transection through Akt activation (Chen et al., 2016a). Src-suppressed protein kinase C substrate (SSeCKS) has been indicated as a suppressor of SC differentiation and myelination by inhibiting Akt activation. SSeCKS is effectively down-regulated during SC differentiation and its depletion promotes Akt phosphorylation and myelin gene expression in cAMP-treated primary cultures or myelination when co-cultured with DRG neurons (Ji et al., 2010). SSeCKS is also essential for SC adhesion, spreading and migration (Yan et al., 2009). In addition, Chen and collaborators showed that cell adhesion molecule 3 (Cadm3) might act as a negative modulator of PNS myelination. Cadm3 interferes with PI3K/Akt activation and inhibits Schwann cell myelination *in vitro* (Chen M. S. et al., 2016).

The mammalian disks large homolog 1 (Dlg1) inhibits PI3K/Akt signaling via its interaction with the phosphatase and tensin homolog deleted on chromosome 10 (PTEN). This mechanism is necessary to transiently inhibit the axonal stimulation of myelination and to limit myelin sheath thickness. Myelin outfoldings and overmyelination followed later by pathologic demyelination are actually observed after Dlg1 silencing via RNA interference in mouse sciatic nerves (Cotter et al., 2010). Dlg1/PTEN functions and consequently Akt/mTOR signaling are also disturbed in different mouse models of demyelinating neuropathies and peripheral nerve sheath tumors (Bolino et al., 2004; Cotter et al., 2010; Goebbels et al., 2010, 2012; Keng et al., 2012). More specifically, Goebbels et al showed that mice lacking PTEN develop a tomaculous neuropathy characterized by focal hypermyelination and myelin outfoldings. Moreover, treating these mice with rapamycin, a mTOR inhibitor, significantly ameliorates the pathology (Goebbels et al., 2010, 2012). Another study on PTEN signaling showed that the hypermyelination observed in mice lacking Dlg1 is actually transitory. This study also identified DNA damage-inducible transcript 4 protein (DDIT4) as a sustained negative regulator of myelination. High DDIT4 expression in SCs precedes the peak of Dlg1 and Akt activity in peripheral nerves. Loss of DDIT4 expression both *in vitro* and *in vivo* results in sustained hypermyelination and enhanced PI3K/Akt/mTOR activation (Noseda et al., 2013). Therefore, it is likely that PI3K/Akt signaling has to be precisely regulated and balanced to induce SC differentiation or dedifferentiation.

mTOR is a signaling pathway that integrates a lot of signals (growth factors, nutrients, energy and oxygen) necessary for growth and proliferation (Norrmén and Suter, 2013). Sherman and collaborators demonstrated that the inactivation of mTOR in SCs provokes an arrest of myelination after axonal sorting and reduces axon growth (Sherman et al., 2012). More recently, it has been reported that the conditional ablation of raptor, an essential component of the mTORC1 complex, in SCs leads to hypomyelination and abnormal lipid biosynthesis (Norrmén et al., 2014). The pathways that converge to mTOR are numerous and include Nrg1/ErbB, PI3K/Akt, MAPKS or AMP-activated kinase (AMPK) signaling (Norrmén and Suter, 2013). AMPK is a heterotrimeric serine/threonine protein kinase acting as an inhibitor of mTOR and described as a crucial energy sensor important to maintain energy homeostasis (Hardie et al., 2012). Recently, Liu and colleagues revealed that AMPK is a critical negative regulator of SC myelination in the PNS (Liu et al., 2016a). They first showed that AMPK is gradually decreased as myelination proceeds. The high activation of AMPK decreases myelin gene expression and stimulates c-Jun expression in SCs whereas its inhibition or depletion induces myelin gene expression. The injection of an inhibitor of AMPK in newborn rats downregulates c-Jun expression, enhances lipid and protein synthesis and increases myelin gene expression and myelin sheath thickness.

### Wnt signaling and LXR

Wnt/β-catenin pathway has been demonstrated to be crucial for PNS development. Cell cultures and genetic experiments revealed that Wnt signaling is required for SC lineage progression, proliferation and myelination (Hari et al., 2002; Lee et al., 2004; Gess et al., 2008; Grigoryan et al., 2013). Recently, it has been proposed that interplay between Wnt pathway and liver X receptors (LXR) induces the fine-tuning of myelin gene expression (Makoukji et al., 2011). Oxysterols acting as ligands for LXR, are natural compounds that originate from the enzymatic oxidation of cholesterol. They are involved in cholesterol homeostasis and in the progression of different neurodegenerative disorders (Lütjohann et al., 2000; Russell, 2000; Leoni et al., 2002). Makoukji and co-workers provided evidence for the implication of LXR and oxysterols in the negative regulation of myelin gene expression (Makoukji et al., 2011). They showed that oxysterols are expressed in a SC line and in sciatic nerves and that they repress the gene expression of *MPZ* and *PMP22* by a mechanism involving LXR α and β and the inhibition of the Wnt/β-catenin-dependent pathway. Unexpectedly, although myelin gene transcripts are up-regulated in mice lacking LXR α and β, presumably due to increased β-catenin activity, myelin protein expression is decreased and myelin sheath thickness is reduced. They conclude that this hypomyelination in the knockout animals may be the consequence of an altered cholesterol homeostasis and an inefficient myelin protein trafficking from the endoplasmic reticulum.

Recently, paraquat, a redox-active herbicide, has been shown to elicit an oxidative stress in the normal sciatic nerves of adult mice and a dramatic disorganization of myelin sheaths, causing severe locomotor and sensory deficits through a stimulation of the LXR pathway leading to reduced Wnt signaling (Hichor et al., 2016). Indeed, the authors provided evidence that paraquat alters myelin gene expression by activating LXR signaling and by preventing β-catenin recruitment on the myelin gene promoter. They also indicated that treating paraquat-exposed mice with lithium, a Wnt/β-catenin modulator acting as a GSK3 inhibitor, prevents the deleterious defects of paraquat on sciatic nerves. The effect of lithium chloride has also been studied after nerve injury. It accelerates axon and myelin debris clearance and remyelination by inhibiting GSK3 and increasing β-catenin levels (Chen et al., 2016b).

### Toll-like receptor signaling

The Toll-like receptors (TLRs) are a class of proteins that play a key role in initiating the immune and inflammatory response. After nerve injury, damage-associated molecular patterns and ligands released from or expressed by activated or necrotic damaged cells result in the activation of TLRs in different cell types including SCs (Thakur et al., 2017). TLR3, TLR4, and TLR7 are constitutively expressed by SCs and TLR1 is upregulated following nerve injury, strongly suggesting a role for TLR signaling in SC-driven nerve repair (Goethals et al., 2010). *In vitro*, the addition of necrotic neurons to SCs activates their inflammatory response through TLR signaling. It increases their gene expression of inflammatory mediators such as tumor necrosis factor-alpha (TNF-α) or monocyte chemoattractant protein-1 (MCP-1) but this effect is reduced in SCs from mice lacking TLR3 (Lee et al., 2006). Boivin and collaborators provided *in vivo* evidence that TLR signaling is involved in WD and nerve regeneration following a nerve injury, possibly through NF-κB activation (Boivin et al., 2007). The early expression of inflammatory modulators such as MCP-1, macrophage recruitment and activation, axonal regeneration and functional recovery are impaired in the mice deficient in TLR signaling. The results are indeed similar in mice lacking TLR2, TLR4 or their adaptor, myeloid differentiation primary response gene 88 (MyD88) known to activate NF-κB. Interestingly, a single injection of TLR2 and TLR4 ligands at the site of the lesion speeds up myelin clearance and functional recovery. Besides its likely role in SC response to nerve injury, TLR signaling has also been linked to the development of neuropathic pain, making TLRs promising therapeutic targets (Thakur et al., 2017).

### GPCR signaling

To date, three different G protein-coupled receptors (GPCRs) have been demonstrated to be implicated in SC differentiation. These are Gpr126, Gpr44, and lysophosphatidic acid receptor 1 (LPA1) (Mogha et al., 2016a). Monk and colleagues were the first ones to show that a GPCR, Gpr126, is crucial for the initiation of SC myelination (Monk et al., 2009). The analysis of mice lacking LPA1 then revealed that this receptor is required for SC migration, axonal sorting and proper myelination (Anliker et al., 2013). Finally, Trimarco et al. provided *in vitro* and *in vivo* evidence for the involvement of Gpr44 and its ligand the prostaglandin D2 in PNS myelination (Trimarco et al., 2014). Very recently, Mogha and collaborators investigated the potentiel functions of Gpr126 in peripheral nerve injury and repair as its expression is maintained in adult SCs (Mogha et al., 2016b). By using an inducible conditional Gpr126 knockout model, they observed defects in remyelination, macrophage recruitment and axon regeneration following a nerve crush. Since approximately 30% of all approved drugs target GPCRs, Gpr126 may represent an attractive potential target to stimulate repair in myelinating disease or after nerve damage.

### Nrg1/ErbB2/3 signaling

Currently, the signals from damaged nerves that induce the reprogramming of SCs are not identified. Some argued that these can be linked to the Nrg1/ErbB pathway, one of the best described interdependent relationship between axons and SCs. Neuregulins form a large family of epidermal growth factor-like proteins that signal through the ErbB tyrosine kinase receptors (Falls, 2003). The Nrg1/ErbB signaling is first of all known for its critical functions in SC development (for review, Newbern and Birchmeier, 2010; Grigoryan and Birchmeier, 2015; Willem, 2016). Indeed, numerous genetic models have been generated to unravel fundamental functions of the Nrg1/ErbB signaling axis. Differential expression of Nrg1 isoforms, their proteolytic processing or ErbB receptor localization and trafficking are important parameters that control the Nrg1/ErbB pathway and its underlying functions. Depending on the developmental stage, the pathway significantly regulates SC survival, proliferation, migration, differentiation and myelination (Syroid et al., 1996; Morris et al., 1999; Leimeroth et al., 2002; Taveggia et al., 2005; Chen et al., 2006; Freidin et al., 2009; Newbern and Birchmeier, 2010). This functional diversity is presumably accomplished through the interaction of Nrg1/ErbB signaling with other pathways. For instance, Notch signaling, which is necessary for SCP proliferation and survival, increases the expression of ErbB2 in SCPs and their sensitivity to Nrg1 (Woodhoo et al., 2009). The tyrosine phosphatase Shp2 is another essential component of the Nrg-1/ErbB pathway that promotes SC proliferation and migration (Grossmann et al., 2009). Later in SC development, critical interactions between Nrg1/ErbB and cAMP or NF-κB signaling are necessary for SC differentiation and myelination (Limpert and Carter, 2010; Arthur-Farraj et al., 2011). Although the importance of Nrg1/ErbB signaling in SC development is well established, its role in adult SC plasticity is more controversial. In adulthood, the pathway seems dispensable for the maintenance of the myelin sheaths, as the absence of Nrg1 in peripheral axons or of ErbB2 in SCs has no effect on axon or myelin sheath integrity (Atanasoski et al., 2006; Fricker et al., 2011, 2013). However, increasing evidence indicates that Nrg1 is necessary for the SC response to nerve injury.

The fact that the Nrg1/ErbB system is selectively and highly regulated during peripheral nerve degeneration and regeneration represents a strong argument in favor of a role for this signaling pathway in SC plasticity (Carroll et al., 1997; Kwon et al., 1997; Ronchi et al., 2016). Recently, a study using a rat surgical model of delayed nerve repair showed that Nrg1 and some SC markers are highly down-regulated after chronic degeneration. Since long-term denervated Schwann cells are known to be partly responsible for delayed nerve repair, it suggests that Nrg1 plays an important function in SC activity after denervation (Ronchi et al., 2017). Guertin and colleagues showed that ErbB2 is activated in myelinating SCs after sciatic nerve axotomy and that its transient activation is sufficient to initiate SC demyelination in compartmentalized cell culture chambers. In addition, the treatment of rats with an ErbB2 inhibitor results in a reduction of demyelination after nerve transection (Guertin et al., 2005). Consistently, Syed et al demonstrated that a robust activation of ErbB2/3 via high concentration of soluble Nrg1 induces SC dedifferentiation and demyelination as well as increased c-Jun expression in primary cultures (Syed et al., 2010). Furthermore, *in vitro* experiments recapitulating nerve injuries provide evidence that Nrg1 treatment shows beneficial effects on SC migration and proliferation (Mahanthappa et al., 1996; Li et al., 1998). Finally, Lee and collaborators showed that Nrg1 on motor axons controls the terminal SC-mediated synapse elimination at the neuromuscular junctions (NMJs) during development. They also revealed that NMJs of adult transgenic mice overexpressing Nrg1 in motor axons exhibited continued remodeling, identifying Nrg1 as a molecular determinant for SC-driven neuromuscular synaptic plasticity (Lee et al., 2016).

Nrg1/ErbB signaling has also been linked to different pathways mentioned earlier for regulating SC plasticity. For example, Nrg1 has been proposed as the signal driving high Erk activation after injury (Napoli et al., 2012). High concentrations of Nrg1 indeed highly activates Erk signaling and induces SC dedifferentiation and demyelination (Syed et al., 2010; Newbern and Snider, 2012). In agreement with this, Tapinos and collaborators also showed that direct binding of Mycobacterium leprae onto ErbB2 receptors on SCs activates Erk signaling and promotes demyelination (Tapinos et al., 2006). Another possibility is that Nrg1 could trigger Rac/JNK activation. Indeed, the inhibition of ErbB2 signaling has been shown to prevent MKK7 activation, c-jun expression, and Rac-dependent gene transcription in cultures of sciatic nerve explants (Shin et al., 2013). ErbB2 also directly binds and activates the negative regulator of myelination, Dock7, upstream of Rac/JNK while the depletion of Dock7 attenuates Nrg1 effects in primary SC cultures (Yamauchi et al., 2008, 2011). Although these data suggest a role for Nrg1/ErbB signaling in SC dedifferentiation, *in vivo* studies revealed contradictory results. Atanasoski et al. showed that lack of ErbB2 in adult SCs does not affect their proliferation and survival after nerve injury, despite reduced levels of phosphorylated MAPK (Atanasoski et al., 2006). In addition, the absence of the axonal Nrg1 following a nerve crush does not disturb SC proliferation associated with nerve degeneration or the clearance of myelin debris by macrophages. Therefore, adult SCs would not require major Nrg1/ErbB signaling for their proliferation and survival after nerve damage, in contrast to what is observed *in vitro* and during development.

While the implication of Nrg1 signaling in the dedifferentiation of SCs is unclear, its involvement in myelination but also in remyelination after nerve injury is now well established. The transmembrane axonal isoforms Nrg1 type III signals in a juxtacrine manner through ErbB2/3 receptors on SCs and determines their myelin thickness, both during development and following a nerve lesion (Michailov et al., 2004; Taveggia et al., 2005; Fricker et al., 2011, 2013). The axonal Nrg1 is rate limiting but not essential for remyelination since compensations are observed at later stages (Fricker et al., 2013). All the same, myelin sheaths are often thinner after injury, suggesting an insufficent simulation by axonal signals (Schröder, 1972). Another mechanism is actually induced in SCs to supplement these poor neuronal cues. Nerve damage triggers the expression of soluble Nrg1 type I by SCs themselves. This particular autocrine and paracrine signaling in SCs is only required after nerve injury and promotes SC survival, redifferentiation and remyelination (Fricker and Bennett, 2011; Stassart et al., 2013).

Besides the importance of Nrg1/ErbB signaling in SC remyelination, it also plays a key role in repair processes after nerve injury (Fricker and Bennett, 2011). Fricker and colleagues showed that the absence of Nrg1 in adult axons after sciatic nerve crush results in defects of remyelination but also in slower axon regeneration, impaired muscle reinnervation and delayed functional recovery (Fricker et al., 2011, 2013). In a mouse model lacking β-Site amyloid precursor protein cleaving enzyme 1 (BACE1), a protein known to cleave and activate Nrg1 type III, SC remyelination is impaired and nerve repair is surprisingly accelerated (Hu et al., 2008). Indeed, knockout mice show an accelerated clearance of axonal and myelin debris from degenerated fibers, faster axonal regeneration and earlier reinnervation of neuromuscular junctions, compared to littermate controls (Farah et al., 2011). The enhanced myelin clearance might clearly be due to the initial hypomyelination observed in adult BACE1 KO sciatic nerves. However, how BACE1 deletion enhances peripheral regeneration still needs to be clarified. Another enzyme capable of cleaving the axonal Nrg1, counteracting BACE1 and interfering with SC differentiation is the tumor necrosis factor-α–converting enzyme (TACE, also named ADAM17). TACE depletion in SC-DRG neuron co-cultures increases myelination. The absence of TACE in motoneurons *in vivo* results in hypermyelination. The function of TACE is neuron autonomous since its knockdown in SCs does not affect myelination. Mechanistically, TACE cleaves Nrg1-III, which is no longer capable of activating PI3K signaling and inducing myelination (La Marca et al., 2011). Therefore, TACE can be considered as a modulator of NRG1 type III activity and as a negative regulator of SC myelination. The role of Nrg1 in peripheral regeneration has also been demonstrated in different studies examining the effects of Nrg1 treatment following nerve injury. The application of exogenous Nrg1 or strategies that elevates Nrg1 levels after nerve damage improve myelin clearance, axon regeneration, remyelination and functional recovery (Chen et al., 1998; Joung et al., 2010; Fricker and Bennett, 2011; Yildiz et al., 2011). Therefore, treatment with recombinant forms of Nrg1 may represent interesting therapeutic avenues to improve nerve repair following injury. It is however necessary to better understand the mechanisms by which Nrg1 treatment improves nerve regeneration.

Finally, it is essential to remember that the inappropriate overactivation of Nrg1/ErbB signaling pathway may be harmful in some circumstances and lead to demyelinating neuropathies or peripheral tumors. For instance, constitutive activation of the Nrg1/ErbB pathway promotes the proliferation of peripheral neuroepithelioma and neoplastic SC line (Frohnert et al., 2003; Fallon et al., 2004). Similarly, transgenic mice over-expressing Nrg1 in myelinating SCs develop hypertrophic neuropathies and malignant peripheral nerve sheath tumors (Huijbregts et al., 2003). In addition, leprosy, which is an important cause of demyelinating neuropathy in the world, is initiated by an excessive activation of ErbB2 by *M. leprae* (Tapinos et al., 2006).

## Pathophysiological implications and therapeutic strategies

Peripheral neuropathies are responsible for significant morbidity and decreased quality of life due to weakness, sensory loss and neuropathic pain. The causes are multiple: peripheral nerve traumatic injuries, inherited genetic diseases such as CMT, metabolic disorders including diabetes, infectious, toxic and inflammatory disorders (Zhou and Notterpek, 2016). Peripheral nerves have an impressive ability to regenerate compared to the CNS. However, the clinical recovery of patients suffering from peripheral neuropathies is often incomplete due to slow regeneration rate, target mis-reinnervation and lack of a long-term regeneration-supportive environment. After injury, peripheral axons regenerate at a very slow rate of approximately 1 mm per day, depending on the lesion site. Moreover, the regenerative capacity of the PNS decreases over time. Indeed, SCs become unsupportive to regeneration after long periods and they also exhibit diminished plasticity with age (Fu and Gordon, 1995; Zochodne, 2012; Painter et al., 2014). Of note, Joshi and coworkers recently demonstrated that SCs show an altered expression of c-Jun and Cdkn1c and that their pro-regenerative functions are diminished in a model of chronic inflammatory demyelinating polyneuropathy (CIDP) (Joshi et al., 2016). A better understanding of the signaling pathways that drive SC reprogramming and plasticity is of great interest and may offer new opportunities to enhance nerve repair. SCs are the main effectors of regeneration in many peripheral diseases and recent studies clearly positioned SCs at the hub for organizing the environment to clear myelin debris, promote axonal regrowth, remyelination and allow complete functional repair following injury (Zochodne, 2012; Kim et al., 2013).

The different signaling pathways and molecular components described in the present review represent interesting targets to boost the endogenous regeneration potential of SCs. For instance, the treatment with different Nrg1 isoforms or strategies to stimulate ErbB signaling have actually been shown to improve myelin clearance, axon regeneration, remyelination and functional recovery after nerve injury in different studies (Chen et al., 1998; Joung et al., 2010; Fricker and Bennett, 2011; Yildiz et al., 2011). In a CMT1A rat model, soluble Nrg1 therapy during early postnatal life prevents abnormal developmental demyelination and subsequent axon loss observed in adulthood (Fledrich et al., 2014). The overexpression of Nrg1 in mouse models of amyotrophic lateral sclerosis (ALS) and partial muscle denervation improves functional recovery by enhancing collateral reinnervation sprouting through both Akt and Erk pathways (Mancuso et al., 2016). Notch activation through the addition of a recombinant jagged1 after injury also promotes nerve regeneration and functional recovery (Wang et al., 2015). The inhibition of mTOR signaling with rapamycin in mice that lack PTEN and develop a tomaculous neuropathy significantly ameliorates the pathology (Goebbels et al., 2012). Finally, stimulating TLR signaling at the site of the lesion accelerates myelin clearance and functional recovery (Boivin et al., 2007).

Additional molecules, not directly linked to pathways analyzed in this review, have been shown to regulate SC differentiation/dedifferentiation and improve nerve repair. For example, Fingolimod, an agonist of the sphingosine-1-phosphate receptor (S1PR), previously known to affect SC migration and myelination *in vitro* and currently in clinical trial for the treatment of CIDP, has been demonstrated to modulate SC plasticity (Köhne et al., 2012; Heinen et al., 2015). Fingolimod activates different dedifferentiation markers such as c-Jun, increases the expression of growth factors, slows myelination and improves axon regeneration. Therefore, Fingolimod supports the generation of a repair promoting cellular phenotype and could be used for the treatment of peripheral nerve damages and diseases (Heinen et al., 2015). Its mechanism of action, however, still remains to be elucidated. Another recent example is the addition of TGF-β1 and forskolin directly on chronically injured nerves that can reactivate the growth-supportive environment through the reprogramming of chronically denervated SCs, the re-induction of their proliferation, and the re-expression of regeneration-associated proteins (Sulaiman and Nguyen, 2016). Immunoglobulins (IVIG) are another therapeutic strategy widely used to treat immune-mediated neuropathies such as CIDP. Tzekova and colleagues demonstrated that IVIG positively influence SC differentiation and maturation and increase their potential to induce axonal outgrowth (Tzekova et al., 2015).

Other approaches that are based on SC regenerative properties and improve nerve repair can also be addressed. First, exercise and electrical stimulation have been demonstrated to promote SC-driven peripheral nerve regeneration in both animal models and in human patients (Gordon and English, 2016). Second, transplantations of SCs from healthy nerves or engineered from different tissues thanks to the emergence of the induced pluripotent stem cell technology are another interesting method to support nerve healing (Zhou and Notterpek, 2016). Actually, numerous SC-based therapies also exist to promote repair in the lesioned CNS (Matsas et al., 2008). Third, there has been major progress in developing biomaterials that improve regeneration by providing trophic factors or by supporting the regenerative functions of transplanted cells *in situ* (Marquardt and Sakiyama-Elbert, 2013). For example, Liu and colleagues recently showed that Salidroside and tissue engineering using SCs and poly lactic-co-glycolic acid promote peripheral nerve regeneration and functional recovery after sciatic nerve transection in rats. Salidroside increases the proliferation and regenerative function of SCs, probably through the modulation of neurotrophic factors (Liu et al., 2017). Fourth, gene therapy that allows to deliver genetic material directly into somatic cells is another option to treat neuropathies that involve gene mutations or to improve nerve regeneration after injuries (Zhou and Notterpek, 2016). In hereditary diseases including CMTs, loss or gain of function mutations could be restored through the re-expression of the absent or non-functional protein or through the down-regulation of the mutant mRNA via RNA interference strategy, respectively. For example, Kagiava and coworkers recently demonstrated that the intrathecal injection of a lentiviral vector with a myelin-specific promoter restored the expression of a neuropathy-associated gene and rescued a model of demyelinating peripheral neuropathy (Kagiava et al., 2016). Also, other studies based on gene therapy in order to improve nerve regeneration used lentiviral vectors that potentiate the therapeutic functions of SCs transplanted in artificial nerves, in autografts or present in damaged nerves (Hoyng et al., 2015). Even if SCs are known to mainly support nerve repair through an enhanced expression of neurotrophic factors that stimulate axon regeneration and myelination, this review highlights the numerous additional roles and regenerative functions of SCs in nerve injuries and in demyelinating diseases. Therefore, targeting the molecular mechanisms that regulate SC reprogramming by gene therapy can be highly promising to stimulate nerve healing. In the future, the challenges will be to optimize gene therapy in order to specifically target SCs and to create safe and regulatable vectors to potentiate their regenerative functions in the appropriate timings. Lipid-based nanoparticles such as liposomes or extracellular vesicles are other valuable tools for the delivery of small molecules or genes that can modulate signaling pathways and promote nerve repair (Takeda and Xu, 2015). In order to selectively introduce material into SCs, Lee and colleagues developed phospholipid-based liposomes that can enter SCs when injected in tail vein and showed that the nanoparticles escape the endogenous degradative mechanisms *in vitro* (Lee et al., 2013). In the following years, it is expected that the improvements in cell and tissue engineering and in approaches that target SC capacity to promote regeneration will lead to the development of innovative and efficient PNS repair strategies.

The different therapeutic options that are built on or that stimulate SC plasticity represent new avenues for nerve regeneration but should be considered with prudence for different reasons. First, a prolonged stimulation of SC dedifferentiation could disrupt the remyelination of the regenerated axons, necessary for rapid nerve conduction and complete functional recovery. Also, the aberrant activation of some pathways including Notch, NF-κB, Ras/Raf/Erk, Rac/JNK, or Nrg1/ErbB can lead to abnormal cell proliferation and the development of peripheral nerve tumors. The better understanding of the molecular mechanisms regulating SC plasticity is thus necessary to set up therapies that modulate both temporally and quantitatively SC capacity to drive nerve repair without affecting remyelination and increasing the chances of triggering tumor development.

## Conclusions

The remarkable capacity of regeneration of the PNS compared to the CNS is mainly due to the fantastic plasticity of SCs. The molecular components involved in the negative regulation of myelination and in the reprogramming of SCs in response to nerve injury provide important mechanistic insights and new therapeutic approaches into peripheral neuropathies as well as in peripheral nerve regeneration. However, this review demonstrates the complexity of the molecular signaling pathways and reveals that little is known about the interactions between them. Moreover, the temporal and quantitative activation of the different signals identified is of great importance but is not completely elucidated. Future studies are thus required to better understand the cross-talks, networks and activation patterns and timings between the different molecular mechanisms controlling SC plasticity.

## Author contributions

Manuscript writing: AB; Manuscript revision: VD, AC, and RF.

### Conflict of interest statement

The authors declare that the research was conducted in the absence of any commercial or financial relationships that could be construed as a potential conflict of interest.

## Abbreviations

ALS: Amyotrophic lateral sclerosis
BACE1: Beta-Site amyloid precursor protein cleaving enzyme 1
BDNF: Brain-derived neurotrophic factor
BMP7: Bone morphogenetic protein 7
Cadm3: Cell adhesion molecule 3
cAMP: Cyclic adenosine monophosphate
Cdkn1c: Cyclin-dependent kinase inhibitor 1C
CIDP: Chronic inflammatory demyelinating polyneuropathy
CMT: Charcot-Marie-Tooth
Cthrc1: Collagen triple helix repeat containing 1
EZH2: Enhancer of zeste homolog 2
Dixdc1: DIX domain containing-1
DDIT4: DNA damage-inducible transcript 4 protein
Dlg1: Disks large homolog 1
Dock7: Dedicator of cytokinesis protein 7
DRG: Dorsal root ganglion
Dusp15: Dual specificity phosphatase 15
Egr-1: Early growth response protein 1; Egr-2/Krox-20Early growth response 2
Egr-3: Early growth response protein 3
Erk: Extracellular signal regulated kinase
GDNF: Glial cell-derived neurotrophic factor
GFAP: Glial fibrillary acidic protein
GPCR: G protein-coupled receptor
Id2: DNA-binding protein inhibitor 2
IVIG: Immunoglobulins
JNK: c-jun N-terminal kinase
LKB1/STK11: Liver kinase B1/ Serine/threonine-protein kinase 11
LPA1: Lysophosphatidic acid receptor 1
LXR: Liver X receptor
MAPK: Mitogen activated protein kinase
MBP: Myelin basic protein
MPZ: Myelin protein zero
MyD88: Myeloid differentiation primary response gene 88
NF-κB: Nuclear factor-kappa B
NMJ: Neuromuscular junction
Notch: Neurogenic locus notch homolog protein
Nrg1: Neuregulin 1
NuRD: Nucleosome remodeling and deacetylase complex
p75NTR: Low affinity neurotrophin receptor
Pax-3: Paired box protein 3
PI 3-kinase: Phosphatidylinositol 3-kinase
PMP22: Peripheral myelin protein 22
Pou3f1/Oct6: POU domain class 3 transcription factor / Octamer-binding factor 6
PTEN: Phosphatase and tensin homolog deleted on chromosome 10
S1PR: Sphingosine-1-phosphate receptor
Sam68: Src-associated in mitosis of 68 kD
Sox2: SRY-related HMG-box gene 2
SSeCKS: Src-suppressed protein kinace C substrate
STK11: Serine/threonine protein kinase
TACE: Tumor necrosis factor-α–converting enzyme
TGFβ: Transforming growth factor beta
Zeb2: Zinc-finger E-box-binding homeobox 2.
